# Editorial: Data perceptualization for climate science communication

**DOI:** 10.3389/fpsyg.2023.1263971

**Published:** 2023-08-11

**Authors:** PerMagnus Lindborg, Shauhrat S. Chopra, Katharina Groß-Vogt

**Affiliations:** ^1^SoundLab, School of Creative Media, City University of Hong Kong, Kowloon, Hong Kong SAR, China; ^2^School of Energy and Environment, City University of Hong Kong, Kowloon, Hong Kong SAR, China; ^3^Institut für Elektronische Musik und Akustik, Kunstuniversität Graz, Graz, Austria

**Keywords:** climate change, science communication, perceptualization, sonification, visualization, multimodal communication, climate

## 1. Introduction

Sonification and visualization are tools for communicating a deeper understanding of the climate crisis and for stimulating action. Technically, they are umbrella terms for the translation of data into sound and light, respectively. They are examples of perceptualization, the study and design of ways to recast scientific knowledge into expressions that inform, engage, and excite. In February 2022, the Guest Editors organized a Conference on Data Art for Climate Action (DACA). The present collection of articles is a natural continuation, aiming to extend and deepen the discussion on sonification and visualization, and to stimulate a renewed look at what these techniques can do for science. How might scientists and designers together approach data art as a means for discovery and communication? Gathered in this Research Topic are four articles that deal with the characterization and systematic translation of climate science into sound and light, music and image, composition and narrative. Reflecting practical, methodological, and theoretical aspects of the topic, it contains a systematic review, a perspective or position paper, a brief research report, and a piece of original research. Each of the articles engages with climate science, communication, data, and action in a different way: via aesthetics and characteristics, artistic affectivization, narrative, and knowledge mapping.

## 2. Research themes

Lindborg et al. present an analysis of topics, qualitative characteristics, and perceived aesthetics in 32 recent sonification and visualization projects that target climate data. The projects were systematically selected from an initial list of almost 400, gathered from the Data Sonification Archive and the Conference on Data Art for Climate Action. The authors gathered textual, graphical, and sonic information about the projects, and created a classification of projects by data source. They developed 25 scales to let raters independently evaluate a range of characteristics, which were then reduced using factor analysis to five essential aspects, labeled Action, Technical, Context, Perspective, and Visualization. In the next step, the Aesthetic Perspective Space (APS), a framework proposed by Paul Vickers and collaborators, was instrumentalized to estimate Intentionality (musical vs. informatical) and Indexicality (concrete vs. abstract) in the projects. Using regression analysis, the authors found evidence of relationships between these variables. The article discusses methods and results, and describe how the dynamics play out in specific projects. There is an ongoing discussion in sound design and auditory perception research communities regarding the relationship between sonification and electroacoustic music composition, which the study contributes toward. It also suggests directions for the development of empirically founded design techniques that might be more effective in serving the communication needs of climate science.

When presented with artistic representations of climate data, audiences might find them persuasive and exciting, which may stimulate positive action, or they might see them as limited or overly conceptual, which may evoke suspicion or boredom. Buening et al. focus on the capacity of data art to not only deal with perceptualization, but also and in parallel, to elicit an “artistic affectivization” that has a potential to lead to perspective-sharing within a community. An artwork is successful if it is able to simultaneously achieve both information-transfer (e.g., an understanding of the science data) and affectivization (e.g., an emotional engagement with the subject matter in its context). The authors propose that a specific psychological mechanism, cognitive dissonance, lies at the heart of a creative strategy that deliberately mixes sugar and spice: to make observers uncomfortable with what they see, and through this reaction provoke self-adjustment and action. If an artwork interpolates between factual and counterfactual expressions, it might create an “ontological instability” or “weirdness” in the mind of the beholder. Ultimately, such dissonance at the conceptual level could trigger an increase in environmental consciousness.

The work of LC et al. seeks to explore this mix of factual and counterfactual information. They start with the observation that people tend to regard a crisis as important only when it becomes a threat to themselves. As a consequence, the authors then seek to explore a novel format of communication to reach alienated audiences: comic fiction. The article reports the background to narrative methods forming a design strategy. They discuss the details of visual comics that they made, wherein everyday human experiences and situations are used to create a relatable story that is in fact an allegory aiming to discuss specific aspects of the climate crisis that are hard to grasp, such as weighing short-term gains against future losses or the altruistic sharing of limited resource. They report results from a questionnaire study of the effectiveness of their visual comics, in terms of the readers learning about climate change, promoting future-directed thinking, and other reactions.

The study by Zong et al. investigates environmental research in Germany using bibliometrics and a visual analysis method to scrape web resources. The authors argue that while German research in the field of climate crisis mitigation is advanced, they find it is not clearly presented for Chinese readers, and that this is a problem given the importance of knowledge transfer from Europe to China, especially in the context of international relations created through the Belt and Road initiative. In this perspective, rendering a “digital portrait” or “visual map” of German environmental research becomes crucial, and the authors argue the usefulness of a visual mapping of knowledge networks.

## 3. Concluding remarks

Considering the broad scope of the call for papers, the topics and research questions addressed by the submissions we received were diverse. Looking retrospectively at the four articles here included, we may identify commonalities between them. For example, the creative practice of LC et al. does indeed create the “ontological instability” that Buening et al. shows to be psychologically effective, and the latter's model for environmental consciousness bears strong resemblance with the latent factors that emerged in the study by Lindborg et al.. While the four articles certainly do not cover the full range of questions being debated in the context of data perceptualization, they do detect a few “hot topics” and areas of interest for researchers and practitioners in the field. Sometimes, as indicated by Zong et al., data perceptualization techniques might have an impact on international policy in the field of climate science and politics. Going forward with new designs, methodologies, and results, it will be essential to include more people in the debate: the public, researchers, practitioners, artists, and professionals with different skills and expertise.

## Author contributions

PL: Writing—original draft, Conceptualization, Investigation. KG-V: Conceptualization, Writing—review and editing. SC: Conceptualization, Writing—review and editing.

